# Polygenic risk scores: en route to clinical practice

**DOI:** 10.1515/medgen-2026-3022

**Published:** 2026-07-08

**Authors:** Markus M. Nöthen, Johannes Schumacher

**Affiliations:** University of Bonn & University Hospital of Bonn Institute of Human Genetics Venusberg-Campus 1 53127 Bonn Germany; Philipps University Marburg & University Hospital of Marburg Institute of Human Genetics Baldingerstr. 35043 Marburg Germany

The idea that common diseases have a polygenic basis is not new. Over a century ago, Fisher demonstrated that the inheritance of quantitative traits is compatible with the joint action of multiple hereditary factors [1]. Nonetheless, this concept has only become operationally tractable over the past two decades, as a result of genome-wide association studies (GWAS) in ever-larger cohorts, and the establishment of biobanks that combine the genetic and phenotypic data of tens of thousands to millions of individuals. Originally, GWAS were undertaken to identify genes and biological pathways underlying common diseases, and they have indeed proven highly productive in this respect [2, 3]. As a by-product of the GWAS approach, however, the effects of multiple variants – each contributing only a small increment to disease risk – can also be aggregated into a single value: the polygenic risk score (PRS) [4, 5].

While the field of PRS research has matured considerably in terms of statistical approaches, clinical translation lags conspicuously behind. Unlike the identification of a new disease gene in a monogenic disorder, which can usually be incorporated into diagnostic practice without substantial delay, clinical translation of a PRS is considerably more demanding. In large part, this is due to the fact that while a PRS provides a good explanation of differences in risk at the population level, what it means for a given individual is much harder to determine. The decisive question is therefore no longer whether such a score can be computed, but whether – and in which contexts – it is of actual clinical benefit to patients.

This thematic issue brings together review articles spanning the arc from methodological foundations through the relevant clinical and regulatory frameworks to the principal fields of application. Each contribution makes the potential of PRS for modern precision medicine apparent, without losing sight of the challenges that must be overcome in order to enable successful clinical translation.

A brief note on terminology: polygenic score (PGS) is the broader, umbrella term, covering the aggregation of allelic effects for any trait, whereas the term polygenic risk score (PRS) is used more specifically in cases where the focus is on disease risk. In this thematic issue, we have aimed to apply these terms consistently. We acknowledge, however, that we have not fully succeeded: the distinction between PGS and PRS is not uniformly maintained in the literature itself, and the individual contributions reflect the terminological conventions established in their respective subfields. We ask the reader’s indulgence where this lack of uniformity is apparent.

The methodological foundations are set out in the contribution by **Klinkhammer et al.** [6]. The authors explain how a score is derived from GWAS summary statistics or from individual-level genotype data, and which procedures have been established to date for variant selection and weighting. At the same time, they describe the limitations of current score construction, and outline future avenues for improving predictive value. These include methodological refinement and the use of more comprehensive datasets, e.g. increased representation of non-European populations and the inclusion of rare variants.

The clinical–regulatory frameworks are set out in the contribution by **Schumacher et al.** [7]. The authors provide a cross-disease overview of the central areas of potential clinical application, ranging from the identification of individuals with an increased disease risk, through support in diagnostics, to the appropriate use of medication (pharmacogenomics). The authors also address the need to establish standards for clinical application, both in terms of methodology and the communication of test results. By placing PRS within the framework of the European In Vitro Diagnostic Medical Devices Regulation [8] and of the German Genetic Diagnostics Act (Gendiagnostikgesetz) [9], the contribution also delineates the regulatory landscape. This regulatory perspective reflects the European, and in particular German, background of the contributing authors; analogous considerations will apply, mutatis mutandis, in other jurisdictions.

The remaining contributions to this thematic issue examine selected, principal areas of application in which substantial steps towards clinical translation have already been taken in recent years, and conclude with a broader conceptual perspective on polygenic architecture per se.

**Li et al.** [10] address cardiovascular diseases. At the time of writing, this is the area with the broadest body of evidence for the clinical utility of PRS, and in which the integration of genetic information into established risk models is being discussed in the most concrete terms. The authors highlight in particular how PRS-informed stratification may identify individuals at substantially elevated cardiovascular risk at an earlier age than is possible on the basis of conventional clinical risk factors alone, thereby opening up a window for timely preventive interventions.

**Tüchler et al.** [11] address oncology. The authors describe an area in which PRS are already being applied in clinical practice, i.e. hereditary breast and ovarian cancer. Here, PRS modify the penetrance of monogenic risk variants, and can thus be incorporated into an established setting of genetically informed risk stratification for early detection. Beyond this, the authors explain how PRS are increasingly being used in risk stratification for further oncological disorders and in the development of tailored screening strategies.

**Frach et al.** [12] address psychiatric disorders – a field that has played a pioneering role in PRS development. The high polygenicity of psychiatric disorders meant that early on, researchers realised that adequate statistical power could only be achieved by pooling international datasets within large research consortia. The high polygenicity also created sustained pressure to develop new statistical genetic methods – pressure that the resulting consortium datasets then made it possible to address. This productive interplay has shaped psychiatric genetic research ever since. Beyond their potential predictive role, the authors explain how polygenic scores have begun to reshape psychiatric nosology per se. The discovery of an extensive genetic overlap between diagnostic categories calls the sharp boundaries of traditional classification systems into question, and lends support to more dimensional, transdiagnostic conceptions of psychiatric illness.

**Winkelmann and Schormair** [13] address neurological disorders, and use restless legs syndrome as a translational model. The authors explain how a combination of an unusually strong single-variant effect – uncommon for a polygenic disorder – and well-established quantitative endophenotypes makes it possible to demonstrate, in exemplary fashion, how genetic susceptibility, objectively measurable intermediate phenotypes, and data collected via wearables in longitudinal assessment can be integrated into multimodal prediction models. The authors place these findings in the context of further neurological conditions – i.e. Parkinson’s disease, Alzheimer’s disease, multiple sclerosis, and epilepsy – and thereby highlight both the evidence supporting the clinical utility of PRS in current neurological practice and the limitations thereof.

**Mathey et al.** [14] address pharmacogenomics and the question of the extent to which a polygenic prediction of therapeutic response and adverse drug reactions can enhance or complement current – mostly monogenic-based – methods of testing. Methodological and ethical considerations particular to pharmacogenomics are also discussed.

A conceptually distinct accent is set by the contribution of **Heyne et al.** [15], which examines the polygenic modification of monogenic disease risk. The article makes clear that current SNP-array-based PRS capture only part of the respective heritability, since they focus on common variants, while rare and low-frequency variants, which account for a substantial proportion of the heritable component of many diseases, remain largely beyond their scope. As sequencing technologies become rapidly more affordable, the prospect of capturing the full complement of trait-related variants in a given individual – and of integrating variants across the full spectrum of allele frequencies, from common to rare, into composite scores – is moving within reach. This development will further blur the seemingly clean distinction between monogenic and multifactorial inheritance, and bring classical and polygenic human genetics into closer dialogue.

For all of the momentum gained in the PRS field, a sober appraisal is warranted. PRS are not oracles of fate. They yield probabilities, not diagnoses, and their value unfolds only in conjunction with clinical, biographical, and environmental factors. Their implementation in clinical practice requires a healthcare context that is capable of acting on the consequences of a given result.

In this regard, a number of key points must be considered, and these have been the subject of repeated critical discussion in recent years [16–19]. First, for most common diseases, the diagnostic gain from a PRS alone remains limited. Second, while combining a PRS with established clinical scores does improve prediction, the effect is often only moderate. A further challenge concerns the scores themselves. For a given phenotype, several PRS – derived in different years and from different underlying datasets – are often in concurrent use. This renders comparison of their results across studies and settings problematic, and recent data indicate that different scores can place the same individual at markedly different positions within the risk distribution. Standardisation, including agreement on which score to apply for a given indication, is therefore an important task for the years ahead. In addition, reporting standards remain inconsistent, randomised trials comparing PRS-informed decisions with standard of care remain sparse, and as long as the underlying data are drawn predominantly from individuals of European ancestry, there is a real risk that scores may aggravate, rather than mitigate, existing inequities in healthcare.

Alongside these clinical considerations, the statistical and conceptual premises on which GWAS and PRS rest are also being revisited in the literature. The additive model presupposes a genetic architecture without appreciable epistasis or gene–environment interaction – a simplification that holds up remarkably well in practice, yet remains conceptually open to challenge [20]. Within-sibship analyses have shown that population-based effect sizes for traits such as height and educational attainment shrink systematically once demographic structure, assortative mating, and indirect genetic effects are taken into account [21]; in other words, what is measured as a ‘genetic effect’ is not solely a direct allelic effect. Furthermore, while the omnigenic model proposed by Boyle, Li, and Pritchard [22] does not alter how a PRS is calculated, it does reshape the interpretive ground on which the score stands, if it is the case that essentially every gene expressed in a relevant cell type contributes to the phenotype through regulatory networks. None of these debates is reason to belittle the field: GWAS and PRS demonstrably work in practice. They are, however, reason to retain the methodological humility that befits a fledgling clinical application.

Experts from the field of human genetics are called upon to take the initiative and to shape the implementation of PRS in clinical practice in a proactive manner, rather than leaving this challenging task to commercial providers or to clinical disciplines without dedicated genetic expertise. This must include an insistence on methodological rigour, with transparent discussion of all limitations; advancing randomised trials of clinical effectiveness; and contributing to the further development of robust, evidence-based regulatory frameworks. The contributions in this thematic issue are intended to provide a solid basis for that effort, while sparking curiosity about a field that is undergoing rapid change.

We wish you an inspiring and thought-provoking read.
